# Management of type 2 diabetes with the dual GIP/GLP-1 receptor agonist tirzepatide: a systematic review and meta-analysis

**DOI:** 10.1007/s00125-022-05715-4

**Published:** 2022-05-17

**Authors:** Thomas Karagiannis, Ioannis Avgerinos, Aris Liakos, Stefano Del Prato, David R. Matthews, Apostolos Tsapas, Eleni Bekiari

**Affiliations:** 1 Clinical Research and Evidence-Based Medicine Unit, Second Medical Department, Aristotle University of Thessaloniki, Thessaloniki, Greece; 2 Diabetes Centre, Second Medical Department, Aristotle University of Thessaloniki, Thessaloniki, Greece; 3 Department of Clinical and Experimental Medicine, Section of Metabolic Diseases and Diabetes, University of Pisa, Pisa, Italy; 4 Harris Manchester College, University of Oxford, Oxford, UK; 5 Oxford Centre for Diabetes, Endocrinology and Metabolism, Churchill Hospital, Oxford, UK

**Keywords:** Dual GIP/GLP-1 receptor agonist, Meta-analysis, Systematic review, Tirzepatide

## Abstract

**Aims/hypothesis:**

Tirzepatide is a novel dual glucose-dependent insulinotropic peptide (GIP) and glucagon-like peptide-1 receptor agonist (GLP-1 RA) currently under review for marketing approval. Individual trials have assessed the clinical profile of tirzepatide vs different comparators. We conducted a systematic review and meta-analysis to assess the efficacy and safety of tirzepatide for type 2 diabetes.

**Methods:**

We searched PubMed, Embase, Cochrane and ClinicalTrials.gov up until 27 October 2021 for randomised controlled trials with a duration of at least 12 weeks that compared once-weekly tirzepatide 5, 10 or 15 mg with placebo or other glucose-lowering drugs in adults with type 2 diabetes irrespective of their background glucose-lowering treatment. The primary outcome was change in HbA_1c_ from baseline. Secondary efficacy outcomes included change in body weight, proportion of individuals reaching the HbA_1c_ target of <53 mmol/mol (<7.0%), ≤48 mmol/mol (≤6.5%) or <39 mmol/mol (<5.7%), and proportion of individuals with body weight loss of at least 5%, 10% or 15%. Safety outcomes included hypoglycaemia, gastrointestinal adverse events, treatment discontinuation due to adverse events, serious adverse events, and mortality. We used version 2 of the Cochrane risk-of-bias tool for randomised trials to assess risk of bias for the primary outcome.

**Results:**

Seven trials (6609 participants) were included. A dose-dependent superiority in lowering HbA_1c_ was evident with all three tirzepatide doses vs all comparators, with mean differences ranging from −17.71 mmol/mol (−1.62%) to −22.35 mmol/mol (−2.06%) vs placebo, −3.22 mmol/mol (−0.29%) to −10.06 mmol/mol (−0.92%) vs GLP-1 RAs, and −7.66 mmol/mol (−0.70%) to −12.02 mmol/mol (−1.09%) vs basal insulin regimens. Tirzepatide was more efficacious in reducing body weight; reductions vs GLP-1 RAs ranged from 1.68 kg with tirzepatide 5 mg to 7.16 kg with tirzepatide 15 mg. Incidence of hypoglycaemia with tirzepatide was similar vs placebo and lower vs basal insulin. Nausea was more frequent with tirzepatide vs placebo, especially with tirzepatide 15 mg (OR 5.60 [95% CI 3.12, 10.06]), associated with higher incidence of vomiting (OR 5.50 [95% CI 2.40, 12.59]) and diarrhoea (OR 3.31 [95% CI 1.40, 7.85]). Odds of gastrointestinal events were similar between tirzepatide and GLP-1 RAs, except for diarrhoea with tirzepatide 10 mg (OR 1.51 [95% CI 1.07, 2.15]). Tirzepatide 15 mg led to higher discontinuation rate of study medication due to adverse events regardless of comparator, while all tirzepatide doses were safe in terms of serious adverse events and mortality.

**Conclusions/interpretation:**

A dose-dependent superiority on glycaemic efficacy and body weight reduction was evident with tirzepatide vs placebo, GLP-1 RAs and basal insulin. Tirzepatide did not increase the odds of hypoglycaemia but was associated with increased incidence of gastrointestinal adverse events. Study limitations include presence of statistical heterogeneity in the meta-analyses for change in HbA_1c_ and body weight, assessment of risk of bias solely for the primary outcome, and generalisation of findings mainly to individuals who are overweight or obese and already on metformin-based background therapy.

*PROSPERO registration no.* CRD42021283449.

**Graphical abstract:**

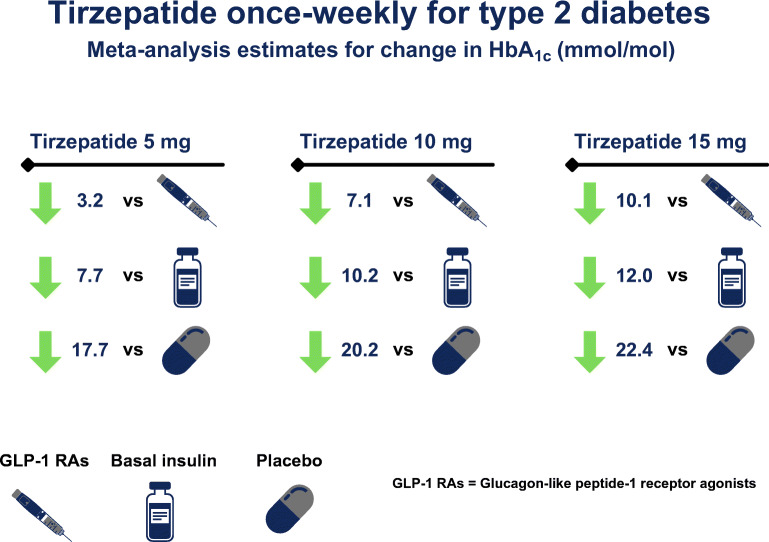

**Supplementary Information:**

The online version contains peer-reviewed but unedited supplementary material available at 10.1007/s00125-022-05715-4.

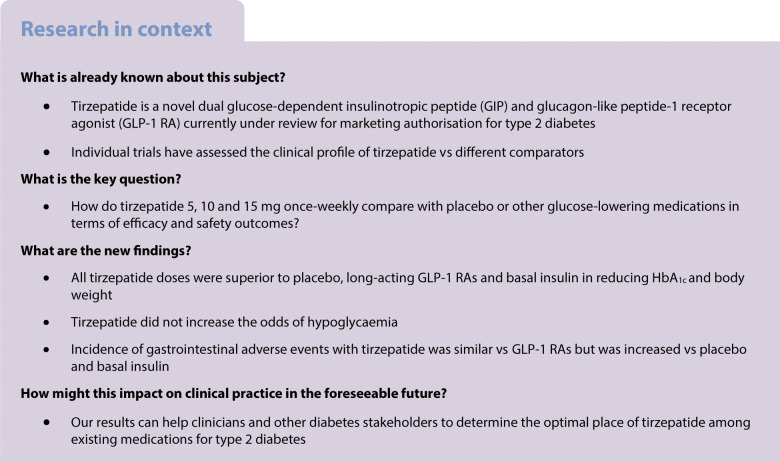



## Introduction

Glucagon-like peptide-1 (GLP-1) and glucose-dependent insulinotropic peptide (GIP) are among the main incretin hormones [1]. GLP-1 is released from L cells in the distal ileum and colon, while GIP is secreted from K cells in the duodenum and jejunum and is responsible for most of the insulinotropic incretin effect [2]. In people with type 2 diabetes the incretin effect is considerably diminished [3]. This has led to the development of GLP-1 receptor agonists (GLP-1 RAs), which have demonstrated favourable effects not only on metabolic variables but also on cardiovascular endpoints [4–6]. The combined GLP-1 and GIP receptor activation has been examined recently as a promising therapeutic concept, given that the two incretins can act on pancreatic beta cells both synergistically and complementarily through distinct metabolic effects [7]. Moreover, GIP can exert therapeutic benefits beyond its primary incretin role, by improving insulin sensitivity and lipid homeostasis in adipose tissue [8].

Tirzepatide (LY3298176) is a dual GIP and GLP-1 RA recently developed for the treatment of type 2 diabetes [9]. It has greater affinity to GIP receptors, rather than GLP-1 receptors, while its t½ of approximately 5 days allows once-weekly subcutaneous administration [9]. Early proof-of-concept and phase 2 studies suggested that tirzepatide can improve both markers of beta cell function and insulin sensitivity compared with selective GLP-1 RA therapy [9, 10]. On the basis of these findings, the overall efficacy and safety of tirzepatide has been investigated in the SURPASS clinical trial programme in comparison with placebo and other glucose-lowering medications including GLP-1 RAs and basal insulin [11]. In a recent news release, the manufacturer announced the submission of a new drug application to the US Food and Drug Administration (FDA) and a marketing authorisation application to the European Medicines Agency (EMA) for tirzepatide for the treatment of adults with type 2 diabetes [12]. We aimed to systematically retrieve all currently available RCTs of tirzepatide in individuals with type 2 diabetes and synthesise the evidence by means of clinically relevant meta-analyses for outcomes of efficacy and safety.

## Methods

The protocol of this systematic review and meta-analysis has been registered in PROSPERO (registration no. CRD42021283449). We report our methods and results in accordance with the Preferred Reporting Items for Systematic reviews and Meta-Analyses (PRISMA) statement [13].

### Eligibility criteria

We included RCTs with a duration of intervention of at least 12 weeks that compared tirzepatide at a maintenance dose of 5, 10 or 15 mg once-weekly, administered subcutaneously, with placebo or any other glucose-lowering medication. Eligible participants were adults with type 2 diabetes irrespective of background glucose-lowering treatment.

### Information sources

We searched PubMed, Embase and Cochrane databases on 27 October 2021 for English-language studies. The search strategy included the keywords ‘tirzepatide’ and ‘ly3298176’ as free-text and MeSH (Medical Subject Headings) terms. Search records that were identified as abstract publications in journals’ supplementary issues of the EASD or the ADA scientific meetings were also considered eligible. We also manually searched the websites of the EASD and ADA scientific meetings, and ClinicalTrials.gov to retrieve either additional eligible trials or any additional information for trials already identified through the database searches.

### Selection process

Results from the databases’ search were imported in a reference management software and, after deduplication, were juxtaposed with the results from the additional search sources. Records were initially screened at title and abstract level, and potentially eligible records were examined in full text with reasons for exclusion being recorded. Two independent reviewers performed the study selection process, and any disagreements were resolved by a third reviewer.

### Data collection process

For each included trial, we used predesigned forms to extract study characteristics, participants’ demographics and baseline characteristics, and outcome data. Our primary outcome was change in HbA_1c_ from baseline. Secondary efficacy outcomes included change in body weight from baseline, proportion of individuals reaching the HbA_1c_ target of <53 mmol/mol (<7.0%), ≤48 mmol/mol (≤6.5%) or <39 mmol/mol (<5.7%) and proportion of patients with at least 5%, 10% or 15% body weight loss. Safety and tolerability outcomes comprised discontinuation of treatment due to adverse events, incidence of serious adverse events, all-cause mortality, hypoglycaemia (plasma glucose ≤3.9 mmol/l), severe hypoglycaemia (a hypoglycaemic event requiring assistance), nausea, vomiting and diarrhoea. The unit of measurement for all dichotomous outcomes was the number of individuals experiencing at least one event of interest. For all outcomes, we extracted data for the modified intention-to-treat population, defined as all randomly assigned participants who received a least one dose of the study drug. For efficacy outcomes, in case trials reported results for different estimand analyses, we preferably extracted data for the efficacy estimand, which represents on-treatment efficacy without the influence of rescue therapy [14]. Data extraction was done by two independent reviewers and arbitrated by a third reviewer.

### Risk-of-bias assessment

We used the version 2 of the Cochrane risk-of-bias tool for randomised trials to assess risk of bias for the primary outcome (change in HbA_1c_) [15]. Overall risk of bias for each trial was considered low if all domains were at low risk of bias, and high if at least one domain was at high risk of bias. In any other case, the risk of bias was deemed as being of some concern. Risk-of-bias assessment was done independently by two reviewers and any disagreements were resolved through consensus. We did not evaluate small-study effect bias with a funnel plot due to the small number of included trials [16].

### Data synthesis

We conducted meta-analyses when at least two studies reported relevant outcome data. For continuous outcomes, we calculated mean differences and 95% CIs using an inverse variance random-effects model. For dichotomous outcomes, we calculated ORs and 95% CIs using the random-effects Mantel–Haenszel approach. In all analyses, we used the Paule–Mandel method to estimate between-study variance [17], and the *I*^2^ statistic to assess statistical heterogeneity. We performed separate analyses based on type of comparator (placebo, GLP-1 RA or basal insulin) and subgroup analyses based on tirzepatide maintenance dose (5, 10 or 15 mg once-weekly). In the placebo-controlled analyses for change in HbA_1c_ and change in body weight, we conducted a post hoc sensitivity analysis excluding one trial with a short duration (12 weeks) [18] and one trial that recruited participants on background insulin therapy [19]. All analyses were done using R version 4.0.5 (R Core Team, Vienna, Austria) and the statistical package ‘meta’.

## Results

### Search results

**Fig. 1:**
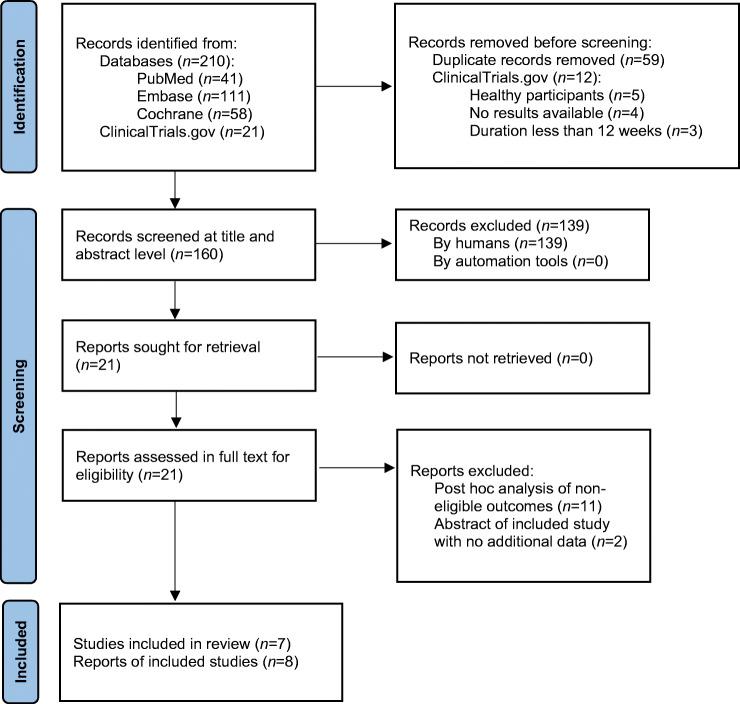
Preferred Reporting Items for Systematic reviews and Meta-Analyses (PRISMA) flow chart for the identification, inclusion and exclusion of studies

The initial search identified 210 results. After screening these records, eight reports of seven RCTs [18–25] with a total of 6609 participants were included in the systematic review and meta-analysis (Fig. 1).

### Study characteristics

The main characteristics of the included studies are presented in Table 1. One study was published in 2022, four in 2021, one in 2020 and one in 2018. Of note, the 2022 study [19] was initially identified in our literature search as a 2021 conference abstract and was subsequently published in a journal during preparation of a revision of our manuscript. Six studies assessed all three eligible tirzepatide maintenance doses (5, 10 and 15 mg once-weekly), while one study included two arms with a maintenance dose of 15 mg but with two different dose-escalation regimens [18]. In our analyses, we merged data for these two arms into a single treatment arm. For one study (SURPASS-2), we used data both from the journal publication [25] and from a conference abstract [24]. The comparator arm was placebo, a GLP-1 RA (subcutaneous administration of semaglutide 1 mg once-weekly) and basal insulin in three [18, 19, 22], one [24, 25] and two trials [20, 21], respectively, while one trial included both a placebo arm and a GLP-1 RA arm (dulaglutide 1.5 mg once-weekly) [23]. All studies had a parallel-group design, and three were open-label. Duration of intervention was 12, 26, 40 and 52 weeks in one, one, three and two studies, respectively. Overall risk of bias for the primary outcome was low in all studies.

**Table 1:** Study-level and participant baseline characteristics of included RCTs

Study; Clinical.Trials.gov registration no.	Study duration, weeks^a^	Blinding status	Background glucose-lowering therapy	Study arms	No. of participants randomised	HbA_1c_, mmol/mol (%)	Body weight, kg	Diabetes duration, years	Age, years
Frias et al, 2018 [23]; NCT03131687	26	Double-blind	Drug naive (9.8%) or metformin monotherapy (90.2%)	Tirzepatide 5 mg	55	66.1 (8.2)	92.8	8.9	57.9
				Tirzepatide 10 mg	51	66.1 (8.2)	92.7	7.9	56.5
				Tirzepatide 15 mg	53	65.0 (8.1)	89.1	8.5	56.0
				Placebo	51	63.9 (8.0)	91.5	8.6	56.6
				Dulaglutide 1.5 mg	54	65.0 (8.1)	89.8	9.3	58.7
Frias et al, 2020 [18]; NCT03311724	12	Double-blind	Drug naive (13.4%) or metformin monotherapy (86.6%)	Tirzepatide 15 mg^b^	56	69.2 (8.5)	89.2	8.6	56.1
				Placebo	26	66.4 (8.2)	89.6	8.8	56.0
Rosenstock et al, 2021 (SURPASS-1) [22]; NCT03954834	40	Double-blind	Drug naive (54%) or previous oral medication use (46%)	Tirzepatide 5 mg	121	63.6 (8.0)	87.0	4.6	54.1
				Tirzepatide 10 mg	121	62.9 (7.9)	86.2	4.9	55.8
				Tirzepatide 15 mg	121	62.3 (7.9)	85.4	4.8	52.9
				Placebo	115	64.5 (8.1)	84.8	4.5	53.6
Frias et al, 2021 (SURPASS-2) [24, 25]; NCT03987919	40	Open-label	Metformin monotherapy (100%)	Tirzepatide 5 mg	470	67.5 (8.3)	92.5	9.1	56.3
				Tirzepatide 10 mg	469	67.2 (8.3)	94.8	8.4	57.2
				Tirzepatide 15 mg	470	66.8 (8.3)	93.8	8.7	55.9
				Semaglutide 1 mg	469	66.7 (8.3)	93.7	8.3	56.9
Ludvik et al, 2021 (SURPASS-3) [20]; NCT038882970	52	Open-label	Metformin monotherapy (68%) or metformin plus SGLT2 inhibitor (32%)	Tirzepatide 5 mg	358	65.8 (8.2)	94.4	8.5	57.2
				Tirzepatide 10 mg	360	65.9 (8.2)	93.8	8.4	57.4
				Tirzepatide 15 mg	359	66.2 (8.2)	94.9	8.5	57.5
				Insulin degludec	360	65.2 (8.1)	94.0	8.1	57.5
Del Prato et al, 2021 SURPASS-4 [21]; NCT03730662	52	Open-label	Monotherapy with or any combination of metformin (95%), sulfonylurea (54%) or SGLT2 inhibitor (25%)	Tirzepatide 5 mg	329	69.6 (8.5)	90.3	9.8	62.9
				Tirzepatide 10 mg	328	70.4 (8.6)	90.6	10.6	63.7
				Tirzepatide 15 mg	338	69.6 (8.5)	90.0	10.4	63.7
				Insulin glargine	1000	69.4 (8.5)	90.2	10.7	63.8
Dahl et al, 2022 (SURPASS-5) [19]; NCT04039503	40	Double-blind	Insulin glargine monotherapy (17%) or in combination with metformin (83%)	Tirzepatide 5 mg	116	67.1 (8.3)	95.5	14.1	61.5
				Tirzepatide 10 mg	119	67.7 (8.3)	95.4	12.6	60.4
				Tirzepatide 15 mg	120	66.4 (8.2)	96.2	13.7	60.5
				Placebo	120	68.2 (8.4)	94.1	12.9	60.0

Data for HbA_1c_, body weight, diabetes duration and age are presented as mean values

^a^Time point at which the primary endpoint was assessed in each study

^b^Data are merged for two trial arms that used two different dose-escalation regimens with tirzepatide 15 mg

SGLT2, sodium–glucose cotransporter 2

### Participant characteristics

Across all trials, participants’ mean HbA_1c_ at baseline was 66.47 mmol/mol (8.2%), mean body weight was 91.5 kg and mean age was 58 years (Table 1). In five studies, background glucose-lowering treatment comprised metformin either as monotherapy or in combination with another oral medication. In one study, more than half the participants (54%) had no previous use of oral glucose-lowering medication [22], while in another study, all participants were on a stable dose of insulin glargine with (83%) or without (17%) metformin [19]. Information regarding the percentage of participants requiring rescue glucose-lowering therapy was retrieved for five studies [20–23, 25]. Based on these data, across all studies, few participants received rescue therapy (ranging approximately between 0.3% and 3.5% of participants), except for one study in which 25% of participants in the placebo arm required rescue therapy [22]. Study medications, GLP-1 RAs, dipeptidyl peptidase 4 inhibitors and pramlintide were not allowed as rescue medications.

### Glycaemic efficacy

**Fig. 2:**
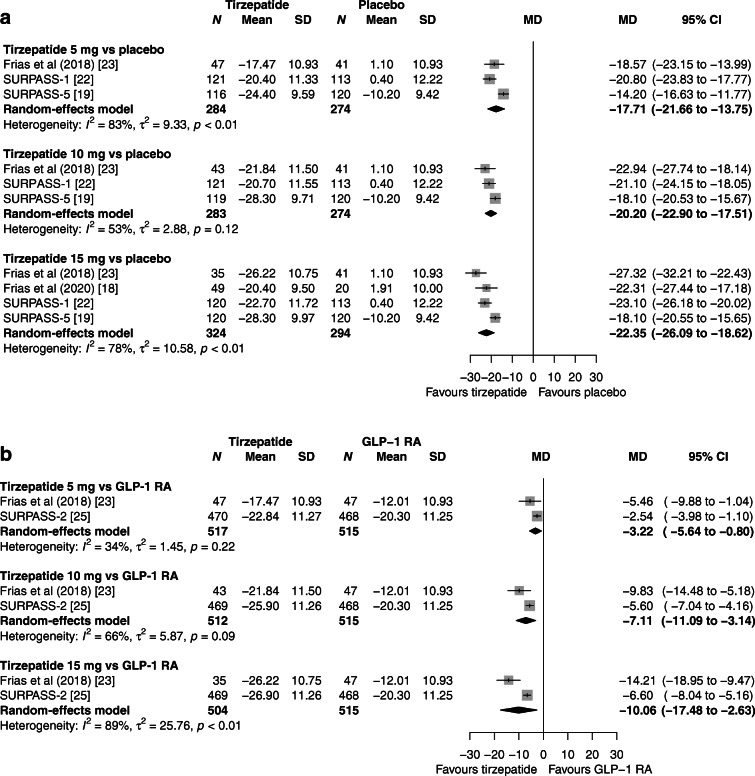
Meta-analysis results for tirzepatide vs placebo (**a**) and vs GLP1-RAs (**b**) for change in HbA_1c_ (mmol/mol). MD, mean difference

Compared with placebo, reductions in HbA_1c_ levels ranged between 17.71 mmol/mol (1.62%) with tirzepatide 5 mg and 22.35 mmol/mol (2.06%) with tirzepatide 15 mg (Fig. 2a). Results were consistent and statistical heterogeneity was reduced in a sensitivity analysis excluding one trial with a short duration and one trial which recruited participants on background insulin therapy. All tirzepatide doses were superior to placebo in terms of achieving the HbA_1c_ target of <53 mmol/mol (<7.0%), ≤48 mmol/mol (≤6.5%) or <39 mmol/mol (<5.7%) (electronic supplementary material [ESM] Table 1). Compared with GLP-1 RAs, tirzepatide 5, 10 and 15 mg reduced HbA_1c_ levels by 3.22 mmol/mol (0.29%), 7.11 mmol/mol (0.65%) and 10.06 mmol/mol (0.92%), respectively (Fig. 2b). Similarly, more participants receiving any tirzepatide dose achieved the three HbA_1c_ targets, except for the target of <53 mmol/mol (<7.0%) with tirzepatide 10 mg, as compared with participants who received a GLP-1 RA (ESM Table 1). All three tirzepatide doses were more effective than basal insulin both in reducing HbA_1c_ (mean differences ranging between 7.66 mmol/mol [0.70%] with tirzepatide 5 mg and 12.02 mmol/mol [1.09%] with tirzepatide 15 mg [ESM Fig. 1]) and in achieving the three HbA_1c_ targets (ESM Table 1). Of note, the mean basal insulin dose at the study endpoint (week 52) was 48.8 U with insulin degludec in the SURPASS-3 trial and 43.5 U with insulin glargine in the SURPASS-4 trial [20, 21].

### Body weight

**Fig. 3:**
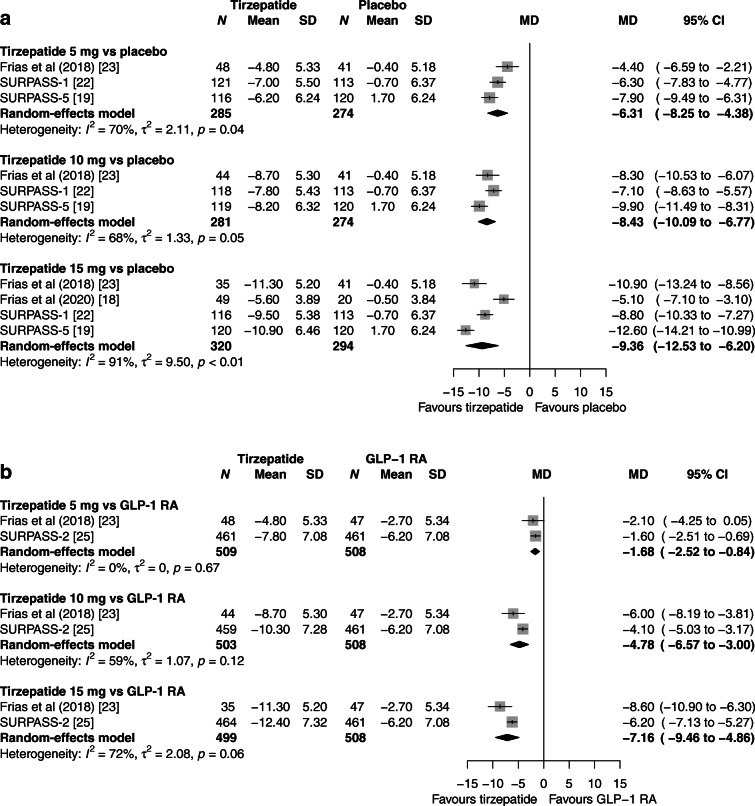
Meta-analysis results for tirzepatide vs placebo (**a**) and vs GLP1-RAs (**b**) for change in body weight (kg). MD, mean difference

Dose-dependent reductions in body weight were evident vs placebo with tirzepatide 5 mg (6.31 kg [95% CI 4.38, 8.25], *I*^2^ 70%), 10 mg (8.43 kg [95% CI 6.77, 10.09], *I*^2^ 68%) and 15 mg (9.36 kg [95% CI 6.20, 12.53], *I*^2^ 91%) (Fig. 3a). The sensitivity analysis excluding one trial with a short duration and one trial that recruited participants on background insulin therapy yielded similar results and reduced statistical heterogeneity. Consistently, compared with placebo, more participants receiving any of the three tirzepatide doses had reductions in body weight of at least 5%, 10% or 15% (ESM Table 2). Tirzepatide induced larger reductions in body weight vs GLP-1 RAs, ranging from 1.68 kg (95% CI 0.84, 2.52 [*I*^2^ 0%]) with tirzepatide 5 mg to 7.16 kg (95% CI 4.86, 9.46 [*I*^2^ 72%]) with tirzepatide 15 mg (Fig. 3b). The OR for achieving a weight loss of at least 5% with tirzepatide 5 mg, 10 mg and 15 mg (vs GLP-1 RAs) was, respectively, 1.96 (95% CI 1.01, 3.80 [*I*^2^ 61%]), 4.79 (95% CI 1.95, 11.73 [*I*^2^ 74%]) and 4.57 (95% CI 3.38, 6.18 [*I*^2^ 0%]) (ESM Table 2). All tirzepatide doses were more efficacious than GLP-1 RAs in achieving a body weight loss of at least 10% and 15% (ESM Table 2). The superiority of tirzepatide in terms of weight control was more pronounced in the comparisons vs basal insulin (ESM Fig. 2 and ESM Table 2).

### Hypoglycaemia

Incidence of any hypoglycaemia (defined as plasma glucose ≤3.9 mmol/l) with tirzepatide did not differ vs placebo (ESM Fig. 3) and was lower with tirzepatide compared with basal insulin (OR ranging from 0.17 with tirzepatide 5 mg to 0.25 with tirzepatide 15 mg) (ESM Fig. 4). We did not pool data for the two studies vs GLP-1 RAs due to the different definition of hypoglycaemia used in each trial. We did not conduct meta-analyses for severe hypoglycaemia because events were rare. Across all trials, a total of 22 participants experienced severe hypoglycaemia, defined as an event requiring assistance: ten with tirzepatide (*n* = 4414); 1 with semaglutide (*n* = 469); and 11 with insulin glargine (*n* = 1000).

### Gastrointestinal adverse events

Compared with placebo, nausea was more frequent with all tirzepatide doses, especially 15 mg (OR 5.60 [95% CI 3.12, 10.06], *I*^2^ 0%) (Table 2). Tirzepatide 15 mg was also associated with higher incidence of vomiting (OR 5.50 [95% CI 2.40, 12.59], *I*^2^ 0%) and diarrhoea (OR 3.31 [95% CI 1.40, 7.85], *I*^2^ 52%), while more participants receiving tirzepatide 10 mg experienced vomiting (OR 2.98 [95% CI 1.13, 7.80], *I*^2^ 0%) (Table 2). Odds of gastrointestinal events were similar between tirzepatide and GLP-1 RAs, except for diarrhoea with tirzepatide 10 mg (OR 1.51 [95% CI 1.07, 2.15], *I*^2^ 0%) (Table 2). Compared with basal insulin, all three tirzepatide doses were associated with dose-dependent increased odds of nausea, vomiting and diarrhoea (Table 2).

**Table 2:** Meta-analysis results for tirzepatide vs placebo, GLP-1 RAs and basal insulin for gastrointestinal adverse events

Intervention	Comparator	No. of participants with outcome/participants analysed		OR (95% CI)	*I*^2^, %
		Tirzepatide arm	Comparator arm		
Nausea					
Tirzepatide 5 mg	Placebo	38/292	13/286	3.02 (1.56, 5.86)	0
	GLP-1 RAs	93/525	100/523	0.91 (0.65, 1.26)	3
	Basal insulin	80/687	29/1360	6.18 (3.93, 9.73)	0
Tirzepatide 10 mg	Placebo	45/291	13/286	3.66 (1.91, 7.02)	0
	GLP-1 RAs	101/520	100/523	1.00 (0.69, 1.45)	11
	Basal insulin	134/688	29/1360	10.93 (5.39, 22.15)	54
Tirzepatide 15 mg	Placebo	82/350	15/312	5.60 (3.12, 10.06)	0
	GLP-1 RAs	125/523	100/523	1.34 (0.99, 1.80)	0
	Basal insulin	161/697	29/1360	13.60 (8.93, 20.72)	0
Vomiting					
Tirzepatide 5 mg	Placebo	15/292	6/286	2.51 (0.95, 6.61)	0
	GLP-1 RAs	31/525	44/523	0.68 (0.42, 1.10)	0
	Basal insulin	37/687	20/1360	3.72 (2.06, 6.72)	0
Tirzepatide 10 mg	Placebo	19/291	6/286	2.98 (1.13, 7.80)	0
	GLP-1 RAs	48/520	44/523	1.11 (0.72, 1.70)	0
	Basal insulin	61/688	20/1360	6.34 (3.69, 10.89)	0
Tirzepatide 15 mg	Placebo	44/350	7/312	5.50 (2.40, 12.59)	0
	GLP-1 RAs	60/523	44/523	1.81 (0.65, 5.08)	68
	Basal insulin	65/697	20/1360	6.66 (3.90, 11.37)	0
Diarrhoea					
Tirzepatide 5 mg	Placebo	39/292	21/286	2.09 (0.77, 5.69)	51
	GLP-1 RAs	75/525	63/523	1.22 (0.85, 1.74)	0
	Basal insulin	96/687	58/1360	3.52 (2.46, 5.05)	0
Tirzepatide 10 mg	Placebo	42/291	21/286	2.26 (0.91, 5.60)	44
	GLP-1 RAs	89/520	63/523	1.51 (1.07, 2.15)	0
	Basal insulin	125/688	58/1360	5.23 (3.74, 7.33)	0
Tirzepatide 15 mg	Placebo	71/350	23/312	3.31 (1.40, 7.85)	52
	GLP-1 RAs	82/523	63/523	1.48 (0.84, 2.64)	39
	Basal insulin	130/697	58/1360	5.59 (4.01, 7.79)	0

### Treatment discontinuation due to adverse events

Discontinuation of treatment due to adverse events did not differ between tirzepatide 5 mg and placebo (OR 1.99 [95% CI 0.83, 4.77], *I*^2^ 0%). However, more participants discontinued treatment with tirzepatide 10 mg (OR 2.39 [95% CI 1.02, 5.59], *I*^2^ 0%) and 15 mg (OR 3.64 [95% CI 1.51, 8.78], *I*^2^ 13%) when compared with placebo (ESM Table 3). Compared with GLP-1 RAs, more participants receiving tirzepatide 15 mg discontinued treatment due to adverse events (OR 2.29 [95% CI 1.39, 3.75], *I*^2^ 0%), whereas no difference was evident for tirzepatide 5 mg and 10 mg (ESM Table 3). Compared with basal insulin, both tirzepatide 5 mg and tirzepatide 15 mg were associated with increased odds of discontinuation of study medication due to adverse events (ESM Table 3).

### Serious adverse events and mortality

Incidence of serious adverse events did not differ between any of the tirzepatide doses and any comparator (ESM Table 3). Across all trials, 41 deaths occurred in individuals receiving tirzepatide (*n* = 4573) and 39 in the comparator arms (*n* = 2151). Of note, 19 of the total deaths were related to Covid-19 disease. We did not pool mortality data in a meta-analysis because most deaths occurred in a single trial which recruited exclusively patients at increased cardiovascular risk (25 deaths with tirzepatide and 35 deaths with insulin glargine) [21].

## Discussion

In this systematic review and meta-analysis we summarised and synthesised the most up-to-date data from RCTs of once-weekly tirzepatide in individuals with type 2 diabetes. Based on our findings, tirzepatide induced dose-dependent reductions in HbA_1c_ that were clinically important, not only vs placebo but also when compared with once-weekly GLP-1 RAs and basal insulin regimens. Notably, this favourable glycaemic effect was not associated with increased risk for hypoglycaemia. With respect to lowering of body weight, a significant dose-dependent effect was evident with tirzepatide even when compared with the GLP-1 RAs semaglutide and dulaglutide. The incidence of gastrointestinal adverse events was similar when comparing tirzepatide with GLP-1 RAs. However, in comparison with placebo or basal insulin, tirzepatide increased odds of nausea, while the doses of 10 and 15 mg were also more likely to cause vomiting or diarrhoea. In addition, treatment with tirzepatide increased odds of discontinuation of study drug due to adverse events. In particular, tirzepatide 15 mg was associated with at least twofold higher odds of study drug discontinuation regardless of comparator. It could be speculated that this increased discontinuation rate vs all comparators, including GLP-1 RAs, may be possibly attributed to the severity of gastrointestinal adverse events experienced with tirzepatide 15 mg, considering that the incidence of gastrointestinal adverse events was similar between tirzepatide and GLP-1 RAs. Finally, tirzepatide was not associated with higher incidence of serious adverse events or all-cause mortality.

Our literature search identified one prior systematic review and meta-analysis with tirzepatide that included four RCTs (2783 participants) [26]. Important differences and methodological considerations render the findings of that meta-analysis non-comparable with our results. More specifically, Bhagavathula and colleagues pooled efficacy outcome data in the same analysis irrespective of type of comparator (placebo or GLP-1 RA) [26]. This introduces clinical heterogeneity and considerably attenuates the practical interpretation of pooled estimates, given the well-established beneficial effects of GLP-1 RAs in reducing both HbA_1c_ and body weight, as opposed to the neutral effect of a placebo intervention [4–6]. Instead, we opted to produce meta-analysis estimates that are clinically relevant and meaningful by conducting separate analyses based on type of comparator (placebo, GLP-1 RAs and basal insulin) for each outcome. Moreover, we included three additional RCTs (two vs basal insulin [20, 21] and one vs placebo [19]), totalling a considerably larger number of participants (*n* = 6609). Additionally, we performed meta-analyses and produced comparative estimates for safety and tolerability outcomes, which are equally important to efficacy measures when deciding on optimal diabetes therapy in clinical practice.

Certain limitations should be considered when interpreting our findings. A degree of statistical heterogeneity, as measured by the *I*^2^ statistic, was present in the analyses for change in HbA_1c_ and body weight. However, heterogeneity was considerably reduced vs placebo in a sensitivity analysis excluding one trial with short duration and one trial in which all participants were on background insulin therapy. Heterogeneity in the analyses vs active comparators could be attributed to differences in efficacy between the two GLP-1 RAs comparators (dulaglutide and semaglutide) or to differences in background glucose-lowering therapy between the two trials with basal insulin. Moreover, we assessed overall risk of bias for each trial solely for the primary outcome of change in HbA_1c_. As such, we did not consider open-label status as a source of bias, given that measurement of HbA_1c_ is an objective outcome and thus is not affected by blinding status [15]. Had we assessed risk of bias for less objective outcomes, such as participant-reported gastrointestinal adverse events, overall risk of bias for such outcomes in these trials might have been deemed of some concern. Furthermore, our results can be generalised mostly to individuals with type 2 diabetes who are already on metformin-based background therapy, given that drug-naive individuals were recruited only in one study [22]. In addition, overall mean body weight of all participants was 91.5 kg and, as such, it is uncertain whether our findings are applicable to individuals with type 2 diabetes who are not overweight or obese. Notably, the effect of tirzepatide as an anti-obesity medication is being investigated in the ongoing SURMOUNT clinical trial programme, in a similar manner to the assessment of semaglutide 2.4 mg for obesity in the Semaglutide Treatment Effect in People with obesity (STEP) programme [27].

In October 2021, the drug manufacturer submitted a marketing authorisation application to the EMA and a priority review voucher to the US FDA for the regulatory approval of tirzepatide in type 2 diabetes, leading to an expected review time of 8 months from the date of submission [12]. As such, tirzepatide is anticipated to receive marketing approval by mid-to-late 2022. Our meta-analysis findings can help clinicians and other diabetes stakeholders to determine the optimal place of tirzepatide among existing medications for type 2 diabetes. We found that tirzepatide is superior in reducing HbA_1c_ compared with other injectable therapies, in particular basal insulin and once-weekly GLP-1 RAs. In addition, tirzepatide, even at the lowest maintenance dose of 5 mg, can reduce body weight to a greater extent compared with GLP-1 RAs including subcutaneous semaglutide which, in turn, has been shown to be superior to other glucose-lowering agents [28]. Notably, head-to-head data for tirzepatide vs GLP1 RAs are available only for dulaglutide 1.5 mg and semaglutide 1 mg. Higher doses of dulaglutide (3.0 mg and 4.5 mg) [29] have also received marketing approval for treatment of type 2 diabetes, while application for a label extension of semaglutide at the dose of 2.0 mg [30] has been submitted to the US FDA and has recently received a positive recommendation by the EMA. At present, even though the comparative efficacy of tirzepatide vs these higher dosing regimens of dulaglutide and semaglutide is unknown, available data suggest that tirzepatide could be a reasonable treatment option when glycaemic control and body weight loss are therapeutic priorities. However, clinicians should also be aware that some individuals receiving tirzepatide may experience gastrointestinal adverse events, which could possibly lead to discontinuation of treatment.

Policy decisions on the reimbursement of tirzepatide in individual countries should be based on health technology assessments integrating long-term efficacy and safety clinical data with country-specific cost-utility analyses comparing tirzepatide with other glucose-lowering medications used in clinical practice. In this regard, it is still unknown whether tirzepatide can induce long-term cardiovascular benefits that are comparable to those of specific GLP-1 RAs or sodium–glucose cotransporter 2 inhibitors [6]. Of note, in the SURPASS-4 trial, adjudicated major adverse cardiovascular events (MACE) were not increased with tirzepatide compared with insulin glargine over an extended follow-up period of 52 additional weeks after the main trial period of 52 weeks [21]. However, the design of SURPASS-4 was based on the change in HbA_1c_ at 52 weeks, and not on MACE, as the primary outcome [21]. The ongoing SURPASS-CVOT trial (ClinicalTrials.gov registration no. NCT04255433) is expected to provide definitive answers on the impact of tirzepatide on cardiovascular disease compared with dulaglutide, a long-acting GLP-1 RA that has been shown to be cardioprotective in individuals with type 2 diabetes at increased cardiovascular risk [31]. Finally, ongoing or recently completed, yet unpublished, RCTs are expected to provide additional information on the comparative effects of tirzepatide vs other glucose-lowering agents, including dulaglutide (ClinicalTrials.gov registration no. NCT03861052), insulin glargine (ClinicalTrials.gov registration no. NCT04093752) and insulin lispro (ClinicalTrials.gov registration no. NCT04537923).

### Conclusions

The findings of this meta-analysis of seven RCTs (6609 participants) suggest a dose-dependent superiority of all three tirzepatide maintenance doses on glycaemic control, not only vs placebo but also vs long-acting GLP-1 RAs and basal insulin regimens. All tirzepatide doses were superior to all comparators in terms of reducing body weight. Treatment with tirzepatide did not increase the odds of hypoglycaemia but was associated with increased incidence of gastrointestinal adverse events, mainly nausea. The dose of 15 mg also increased the odds of discontinuation due to adverse events by at least twofold regardless of comparator. These findings are mostly applicable to individuals on metformin-based background therapy, while further trial data are required to determine whether the salutary metabolic effects of tirzepatide translate to long-term cardiovascular benefits.

## Supplementary Information

ESM (PDF 443 kb)

## Data Availability

The datasets generated during and/or analysed during the current study are available from the corresponding author on reasonable request.

## Abbreviations

EMA: European Medicines Agency
FDA: Food and Drug Administration
GIP: Glucose-dependent insulinotropic peptide
GLP-1 RA: Glucagon-like peptide-1 receptor agonist
MACE: Major adverse cardiovascular events
