# Supplementary material for: Management of type 2 diabetes with the dual GIP/GLP-1 receptor agonist tirzepatide: a systematic review and meta-analysis
Source: Diabetologia. 2022 May 17;65(8):1251–61. doi: 10.1007/s00125-022-05715-4 (PMC9112245; doi:10.1007/s00125-022-05715-4)

Electronic Supplementary Material (ESM) contents

ESM Table 1 Meta-analysis results for tirzepatide versus placebo, GLP-1 RAs, and basal insulin for achievement of different HbA_{1c} targets

ESM Table 2 Meta-analysis results for tirzepatide versus placebo, GLP-1 RAs, and basal insulin for achievement of body weight loss of at least 5%, 10%, and 15%

ESM Table 3 Meta-analysis results for tirzepatide versus placebo, GLP-1 RAs, and basal insulin for discontinuation due to adverse events and for incidence of serious adverse events

ESM Fig. 1 Meta-analysis results for tirzepatide versus basal insulin for change in HbA_{1c} (mmol/mol)

ESM Fig. 2 Meta-analysis results for tirzepatide versus basal insulin for change in body weight (kg)

ESM Fig. 3 Meta-analysis results for tirzepatide versus placebo for incidence of hypoglycaemia

ESM Fig. 4 Meta-analysis results for tirzepatide versus basal insulin for incidence of hypoglycaemia

ESM Table 1 Meta-analysis results for tirzepatide versus placebo, GLP-1 RAs, and basal insulin for achievement of different HbA_{1c} targets

Intervention	Comparator	Participants with outcome/analysed, n		Odds ratio (95% CI)	I^2, %
		Tirzepatide arm	Comparator arm		
**HbA_{1c} < 53 mmol/mol (< 7%)**					
Tirzepatide 5 mg	Placebo	236/292	62/284	16.25 (8.98 to 29.41)	53
	GLP-1 RAs	432/516	402/515	1.47 (1.07 to 2.02)	0
	Basal insulin	555/681	711/1331	3.51 (2.49 to 4.94)	55
Tirzepatide 10 mg	Placebo	250/286	62/284	30.70 (10.14 to 92.98)	83
	GLP-1 RAs	454/509	402/515	4.08 (0.74 to 22.41)	87
	Basal insulin	597/666	711/1331	6.92 (5.25 to 9.12)	0
Tirzepatide 15 mg	Placebo	279/338	64/304	20.87 (10.20 to 42.74)	67
	GLP-1 RAs	469/517	402/515	2.84 (1.96 to 4.11)	0
	Basal insulin	630/686	711/1331	9.02 (6.70 to 12.14)	0
**HbA_{1c} ≤ 48 mmol/mol (≤ 6.5%)**					
Tirzepatide 5 mg	Placebo	214/292	29/284	28.72 (10.71 to 77.00)	69
	GLP-1 RAs	376/516	326/515	1.80 (1.00 to 3.24)	56
	Basal insulin	467/681	466/1331	3.63 (2.71 to 4.87)	52
Tirzepatide 10 mg	Placebo	232/286	29/284	44.95 (14.89 to 135.67)	55
	GLP-1 RAs	418/509	326/515	3.77 (1.28 to 11.08)	81
	Basal insulin	525/666	466/1331	6.26 (5.02 to 7.81)	0
Tirzepatide 15 mg	Placebo	223/289	29/284	35.66 (16.43 to 77.39)	56
	GLP-1 RAs	435/517	326/515	3.19 (2.29 to 4.43)	5
	Basal insulin	572/686	466/1331	8.47 (6.70 to 10.70)	0
**HbA_{1c} < 39 mmol/mol (< 5.7%)**					
Tirzepatide 5 mg	Placebo	69/292	5/284	11.70 (1.93 to 70.75)	56
	GLP-1 RAs	137/516	92/515	1.69 (1.25 to 2.28)	0
	Basal insulin	166/681	52/1331	7.43 (5.33 to 10.36)	0
Tirzepatide 10 mg	Placebo	93/286	5/284	25.48 (10.09 to 64.37)	0

	GLP-1 RAs	214/509	92/515	3.95 (1.64 to 9.50)	27
	Basal insulin	240/666	52/1331	12.80 (9.26 to 17.69)	0
Tirzepatide 15 mg	Placebo	138/289	5/284	45.76 (18.21 to 114.99)	0
	GLP-1 RAs	252/517	92/515	7.13 (1.53 to 33.17)	61
	Basal insulin	315/686	52/1331	19.53 (14.20 to 26.86)	0
CI, confidence interval; GLP-1 RAs, Glucagon-like peptide-1 receptor agonists					

ESM Table 2 Meta-analysis results for tirzepatide versus placebo, GLP-1 RAs, and basal insulin for achievement of body weight loss of at least 5%, 10%, and 15%

Intervention	Comparator	Participants with outcome/analysed, n		Odds ratio (95% CI)	I^2, %
		Tirzepatide arm	Comparator arm		
**Weight loss ≥ 5%**					
Tirzepatide 5 mg	Placebo	161/292	22/284	14.49 (8.63 to 24.33)	0
	GLP-1 RAs	342/516	282/515	1.96 (1.01 to 3.80)	61
	Basal insulin	438/679	100/1337	23.16 (15.47 to 34.67)	50
Tirzepatide 10 mg	Placebo	193/288	22/284	28.58 (10.22 to 79.94)	24
	GLP-1 RAs	414/510	282/515	4.79 (1.95 to 11.73)	74
	Basal insulin	552/670	100/1337	60.01 (36.63 to 98.29)	60
Tirzepatide 15 mg	Placebo	207/289	22/284	33.62 (14.02 to 80.60)	45
	GLP-1 RAs	433/517	282/515	4.57 (3.38 to 6.18)	0
	Basal insulin	595/686	100/1337	84.02 (50.65 to 139.40)	58
**Weight loss ≥ 10%**					
Tirzepatide 5 mg	Placebo	69/292	2/284	34.31 (9.54 to 123.40)	0
	GLP-1 RAs	174/516	122/515	1.65 (1.26 to 2.18)	0
	Basal insulin	249/679	25/1337	28.24 (16.62 to 47.99)	34
Tirzepatide 10 mg	Placebo	114/288	2/284	74.04 (20.85 to 262.92)	0
	GLP-1 RAs	263/510	122/515	3.68 (2.30 to 5.88)	23
	Basal insulin	365/670	25/1337	58.52 (36.31 to 94.32)	20
Tirzepatide 15 mg	Placebo	126/289	2/284	87.42 (24.58 to 311.00)	0
	GLP-1 RAs	321/517	122/515	5.46 (4.15 to 7.18)	0
	Basal insulin	464/686	25/1337	102.68 (66.91 to 157.56)	0
**Weight loss ≥ 15%**					
Tirzepatide 5 mg	Placebo	26/292	0/284	16.72 (3.10 to 90.09)	0
	GLP-1 RAs	73/516	41/515	1.91 (1.28 to 2.87)	0
	Basal insulin	89/679	5/1337	35.16 (14.51 to 85.18)	0
Tirzepatide 10 mg	Placebo	58/288	0/284	46.81 (9.10 to 240.91)	0

	GLP-1 RAs	138/510	41/515	5.00 (1.95 to 12.86)	29
	Basal insulin	176/670	5/1337	73.53 (28.05 to 192.76)	5
Tirzepatide 15 mg	Placebo	76/289	0/284	64.61 (12.56 to 332.32)	0
	GLP-1 RAs	198/517	41/515	7.18 (4.97 to 10.37)	0
	Basal insulin	272/686	5/1337	137.20 (49.17 to 382.79)	8
CI, confidence interval; GLP-1 RAs, Glucagon-like peptide-1 receptor agonists					

ESM Table 3 Meta-analysis results for tirzepatide versus placebo, GLP-1 RAs, and basal insulin for discontinuation due to adverse events and for incidence of serious adverse events

Intervention	Comparator	Participants with outcome/analysed, n		Odds ratio (95% CI)	I^2, %
		Tirzepatide arm	Comparator arm		
**Discontinuation due to adverse events**					
Tirzepatide 5 mg	Placebo	16/292	8/286	1.99 (0.83 to 4.77)	0
	GLP-1 RAs	33/525	25/523	1.34 (0.78 to 2.29)	0
	Basal insulin	62/687	59/1360	3.08 (1.34 to 7.06)	61
Tirzepatide 10 mg	Placebo	19/291	8/286	2.39 (1.02 to 5.59)	0
	GLP-1 RAs	43/520	25/523	1.23 (0.30 to 5.09)	72
	Basal insulin	65/688	59/1360	3.45 (0.72 to 16.58)	89
Tirzepatide 15 mg	Placebo	35/350	9/312	3.64 (1.51 to 8.78)	13
	GLP-1 RAs	53/523	25/523	2.29 (1.39 to 3.75)	0
	Basal insulin	75/697	59/1360	3.99 (1.00 to 15.98)	86
**Serious adverse events**					
Tirzepatide 5 mg	Placebo	15/292	15/286	1.00 (0.47 to 2.12)	0
	GLP-1 RAs	34/525	16/523	1.23 (0.17 to 9.12)	67
	Basal insulin	77/687	215/1360	0.94 (0.51 to 1.75)	71
Tirzepatide 10 mg	Placebo	18/291	15/286	1.22 (0.59 to 2.50)	0
	GLP-1 RAs	28/520	16/523	1.80 (0.96 to 3.39)	0
	Basal insulin	74/688	215/1360	0.84 (0.63 to 1.13)	0
Tirzepatide 15 mg	Placebo	12/350	15/312	0.80 (0.37 to 1.75)	0
	GLP-1 RAs	29/523	16/523	1.65 (0.64 to 4.26)	27
	Basal insulin	67/697	215/1360	0.80 (0.39 to 1.63)	77
CI, confidence interval; GLP-1 RAs, Glucagon-like peptide-1 receptor agonists					

ESM Fig. 1 Meta-analysis results for tirzepatide versus basal insulin for change in HbA_{1c} (mmol/mol).

MD, mean difference

	Tirzepatide			Basal insulin				
**Study**	**N**	**Mean**	**SD**	**N**	**Mean**	**SD**	**MD**	**95% CI**
**A.Tirzepatide 5 mg vs basal insulin**								
SURPASS−3 [20]	358	−21.10	9.46	359	−14.60	9.47	−6.50	(−7.89 to −5.11)
SURPASS−4 [21]	326	−24.50	10.65	978	−15.70	10.63	−8.80	(−10.13 to −7.47)
**Random effects model**	**684**			**1337**			**−7.66**	**(−9.91 to −5.40)**
Heterogeneity: I^2 = 82%, τ^2 = 2.16, p = 0.02								
**B.Tirzepatide 10 mg vs basal insulin**								
SURPASS−3 [20]	360	−24.00	11.38	359	−14.60	9.47	−9.40	(−10.93 to −7.87)
SURPASS−4 [21]	321	−26.60	10.57	978	−15.70	10.63	−10.90	(−12.23 to −9.57)
**Random effects model**	**681**			**1337**			**−10.20**	**(−11.67 to −8.73)**
Heterogeneity: I^2 = 52%, τ^2 = 0.59, p = 0.15								
**C.Tirzepatide 15 mg vs basal insulin**								
SURPASS−3 [20]	358	−26.00	11.35	359	−14.60	9.47	−11.40	(−12.93 to −9.87)
SURPASS−4 [21]	334	−28.20	10.78	978	−15.70	10.63	−12.50	(−13.83 to −11.17)
**Random effects model**	**692**			**1337**			**−12.02**	**(−13.09 to −10.95)**
Heterogeneity: I^2 = 11%, τ^2 = 0.07, p = 0.29								

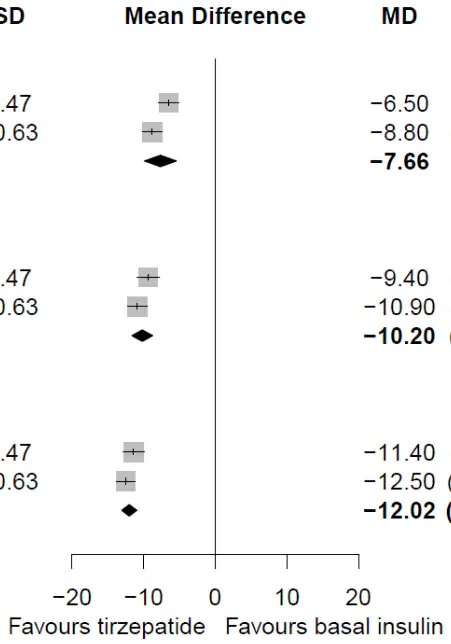

ESM Fig. 2 Meta-analysis results for tirzepatide versus basal insulin for change in body weight (kg).

MD, mean difference

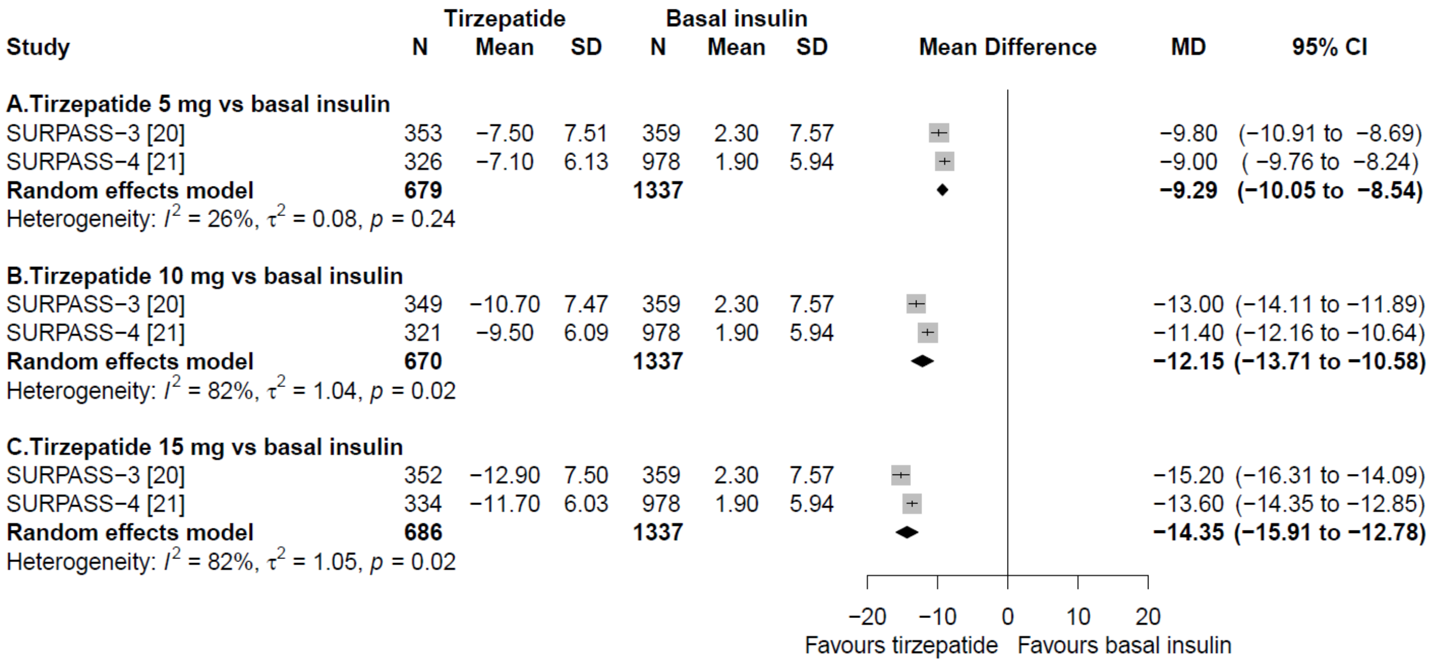

ESM Fig. 3 Meta-analysis results for tirzepatide versus placebo for incidence of hypoglycaemia

Study or Subgroup	Tirzepatide Events	Tirzepatide Total	Control Events	Control Total	Odds Ratio MH, Random, 95% CI
**A.Tirzepatide 5 mg vs placebo**					
Frias et al (2018) [23]	4	55	2	51	1.92 (0.34 to 10.97)
SURPASS−1 [22]	7	121	1	115	7.00 (0.85 to 57.81)
SURPASS−5 [19]	70	116	73	120	0.98 (0.58 to 1.65)
**Total (95% CI)**		**292**		**286**	**1.60 (0.59 to 4.39)**
Heterogeneity: $Tau^2 = 0.37$; $Chi^2 = 3.51$, df = 2 (P = 0.17); $I^2 = 43\%$					
**B.Tirzepatide 10 mg vs placebo**					
Frias et al (2018) [23]	5	51	2	51	2.66 (0.49 to 14.41)
SURPASS−1 [22]	8	121	1	115	8.07 (0.99 to 65.58)
SURPASS−5 [19]	75	119	73	120	1.10 (0.65 to 1.85)
**Total (95% CI)**		**291**		**286**	**1.97 (0.68 to 5.68)**
Heterogeneity: $Tau^2 = 0.44$; $Chi^2 = 4.01$, df = 2 (P = 0.13); $I^2 = 50\%$					
**C.Tirzepatide 15 mg vs placebo**					
Frias et al (2018) [23]	4	53	2	51	2.00 (0.35 to 11.43)
Frias et al (2020) [18]	10	56	0	26	18.99 (0.54 to 670.66)
SURPASS−1 [22]	8	121	1	115	8.07 (0.99 to 65.58)
SURPASS−5 [19]	72	120	73	120	0.97 (0.58 to 1.62)
**Total (95% CI)**		**350**		**312**	**2.27 (0.71 to 7.31)**
Heterogeneity: $Tau^2 = 0.68$; $Chi^2 = 6.49$, df = 3 (P = 0.09); $I^2 = 54\%$					

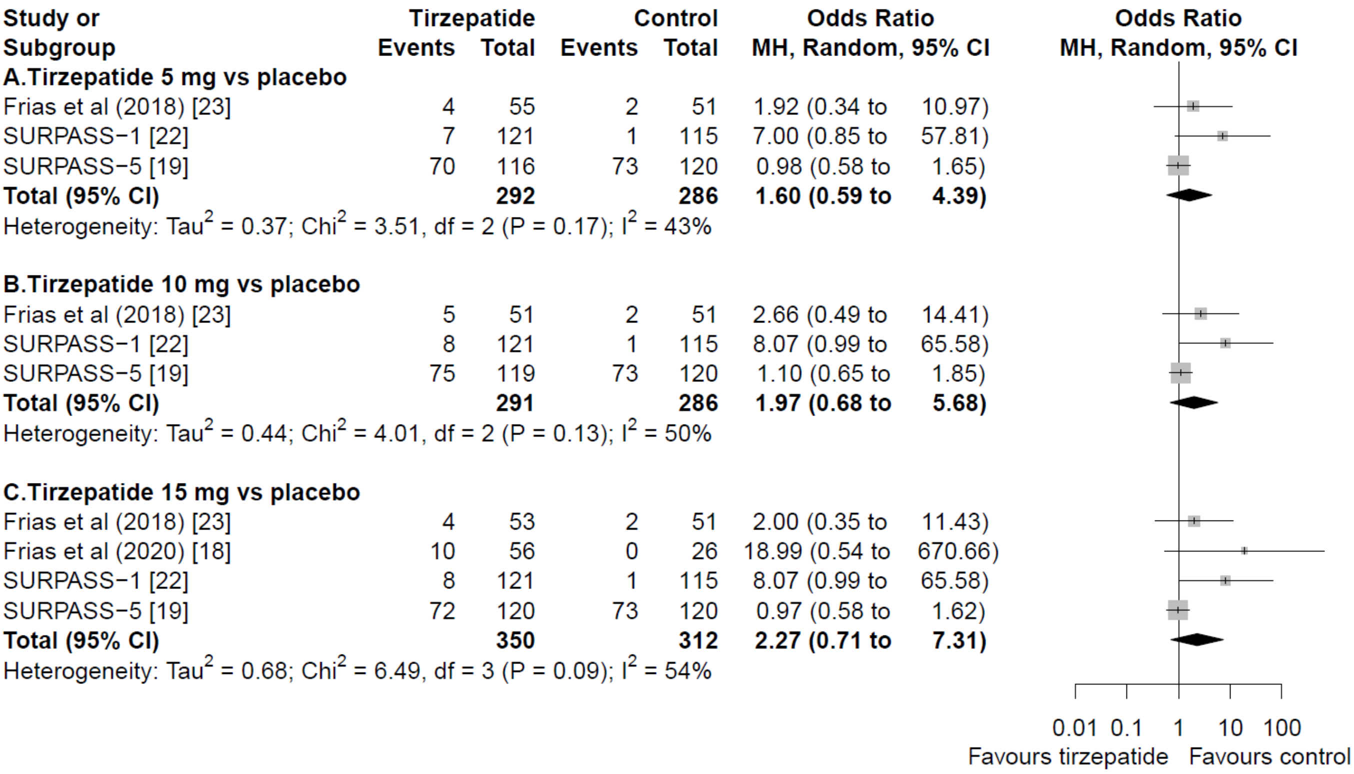

ESM Fig. 4 Meta-analysis results for tirzepatide versus basal insulin for incidence of hypoglycaemia

Study or Subgroup	Tirzepatide Events	Tirzepatide Total	Control Events	Control Total	Odds Ratio MH, Random, 95% CI
**A.Tirzepatide 5 mg vs basal insulin**					
SURPASS−3 [20]	30	358	170	360	0.10 (0.07 to 0.16)
SURPASS−4 [21]	112	329	641	1000	0.29 (0.22 to 0.38)
**Total (95% CI)**		**687**		**1360**	**0.17 (0.06 to 0.48)**
Heterogeneity: Tau^2 = 0.51; Chi^2 = 16.52, df = 1 (P < 0.01); I^2 = 94%					
**B.Tirzepatide 10 mg vs basal insulin**					
SURPASS−3 [20]	49	360	170	360	0.18 (0.12 to 0.25)
SURPASS−4 [21]	107	328	641	1000	0.27 (0.21 to 0.35)
**Total (95% CI)**		**688**		**1360**	**0.22 (0.15 to 0.34)**
Heterogeneity: Tau^2 = 0.07; Chi^2 = 3.52, df = 1 (P = 0.06); I^2 = 72%					
**C.Tirzepatide 15 mg vs basal insulin**					
SURPASS−3 [20]	51	359	170	360	0.19 (0.13 to 0.27)
SURPASS−4 [21]	127	338	641	1000	0.34 (0.26 to 0.44)
**Total (95% CI)**		**697**		**1360**	**0.25 (0.14 to 0.46)**
Heterogeneity: Tau^2 = 0.15; Chi^2 = 7.06, df = 1 (P < 0.01); I^2 = 86%					

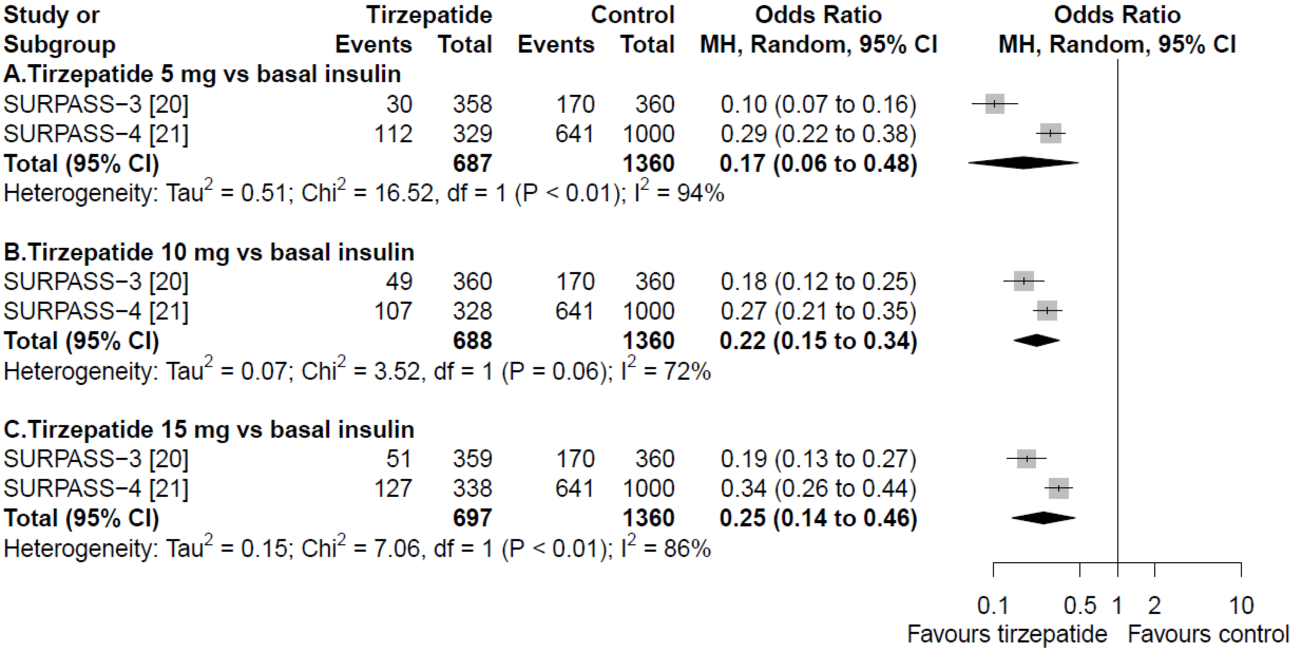

Supplement: Supplementary file 1 — (PDF 443 kb) [file 125_2022_5715_MOESM1_ESM.pdf]
